# Coevolutionary Dynamics with Global Fields

**DOI:** 10.3390/e24091239

**Published:** 2022-09-03

**Authors:** Mario G. Cosenza, José L. Herrera-Diestra

**Affiliations:** 1 School of Physical Sciences & Nanotechnology, Universidad Yachay Tech, Urcuquí 100115, Ecuador; 2 Department of Integrative Biology, University of Texas at Austin, Austin, TX 78712, USA; 3 Centro de Simulacion y Modelos (CeSiMo), Universidad de Los Andes, Mérida 5101, Venezuela

**Keywords:** coevolutionary dynamics, global interactions, adaptive networks, opinion formation

## Abstract

We investigate the effects of external and autonomous global interaction fields on an adaptive network of social agents with an opinion formation dynamics based on a simple imitation rule. We study the competition between global fields and adaptive rewiring on the space of parameters of the system. The model represents an adaptive society subject to global mass media such as a directed opinion influence or feedback of endogenous cultural trends. We show that, in both situations, global mass media contribute to consensus and to prevent the fragmentation of the social network induced by the coevolutionary dynamics. We present a discussion of these results in the context of dynamical systems and opinion formation dynamics.

## 1. Introduction

Many physical, chemical, biological, social, and economic systems are subject to global interactions. A global interaction in a system occurs when all its constituents share a common influence or source of information [1]. The origin of a global interaction can be either external, as in a forcing field; or autonomous, such as a mean field or a feedback coupling function that depends on the elements of the system [2,3]. Global interactions appear, for example, in parallel electric circuits, coupled oscillators [4,5], Josephson junction arrays [6], charge density waves [7], multimode lasers [8], neural networks, evolution models, ecological systems [9], social networks [10], economic exchange [11], mass media influence [12,13,14], and cultural globalization [15]. A complete graph or fully connected network, where any node can interact each each other, can be seen as a global interaction. Diverse collective behaviors can emerge in globally coupled oscillators, such as complete and generalized chaos synchronization, dynamical clustering, nontrivial collective behavior, chaotic itinerancy, quorum sensing, and chimera states [16,17,18,19,20,21,22,23,24,25]. Systems possessing coexisting global and local interactions have also been studied [26].

Most of the research on the effects of global interaction fields has been conducted considering the evolution of the states of the nodes on a fixed network. However, many complex systems observed in nature can be described as dynamical networks of interacting elements where the states of the elements and their connections influence each other and evolve simultaneously [27,28,29,30,31]. The terms coevolutionary dynamical system and adaptive network [29,30] have been employed for systems exhibiting this coupling between the network topology and node state dynamics. Coevolution models have been studied in spatiotemporal dynamical systems, such as neural networks [32,33], coupled map lattices [34,35], motile elements [36], synchronization in networks [37], as well as in spin dynamics [38], epidemic propagation [39,40,41], game theory [27,29,42], and also in the context of social dynamics, such as opinion formation and cultural polarization [43,44,45,46,47,48,49,50]. Coevolutionary systems usually exhibit a transition between two network configurations: a large connected graph where most nodes share the same state, and a fragmented network of small disconnected components, each composed by nodes in a common state [43,44,45,46,47,48,49,50]. This network fragmentation transition is related to the difference in time scales of the processes that characterize the two dynamics: the state of the nodes and the network of interactions [43].

In this article we investigate the effects of global interaction fields on coevolutionary dynamics. We study the competition between adaptive rewiring and global interactions in a network. We consider external global fields or autonomous global fields acting on an adaptive network of social agents with an opinion formation dynamics based on a simple imitation rule. In the context of social phenomena, our system can be considered as a model for a society subject to global mass media that represent a directed opinion influence or a feedback of endogenous cultural trends. We show that, in both situations, global mass media contribute to consensus and to inhibit the fragmentation of the social network. We present a discussion of these results.

## 2. Materials and Methods

We considered a population of *N* social agents represented as nodes on an initially random network of the Erdos–Renyi type with an average degree 〈k〉, i.e., 〈k〉 is the average number of edges per node [51]. We denoted by $\nu_{i}$ the set of $k_{i}$ neighbors of node *i* (i=1,2,…,N). We let $g_{i}$ be the state variable or the opinion of agent *i*, where $g_{i}$ can take any of the *G* equivalent options in the set {1,2,…,G}; i.e., we assumed that the states of the nodes were discrete.

We introduced a global field Φ that could interact with all the elements in the system and that had a state $g_{\Phi }\in \left\{1,2,\ldots ,G\right\}$. The global field could be interpreted as an additional neighbor shared by each node *i* with whom an interaction was possible. Then, the network subject to the global field Φ corresponded to a dynamical system possessing both local and global interactions.

We considered two types of global fields Φ:

(i) An *external global field* whose value $g_{\Phi }$ was chosen from the set {1,…,G} and remained fixed during the evolution of the system. The external field corresponded to a constant spatially uniform influence acting on the system. A constant external field can be interpreted as a specific state (such as an opinion, a message, or advertisement) being transmitted by mass media over all the elements of a social system.

(ii) An *autonomous global field* whose value $g_{\Phi }$ depended on the state variables of the elements in the system. Here, we defined $g_{\Phi }$ as the statistical mode of the distribution of state variables of the agents in the system at a given time, denoted as $g_{\Phi }=\mathrm{mode}\left\{g_{1},g_{2},g_{3},\ldots ,g_{N}\right\}$. That is, we assigned $g_{\Phi }$ as the most abundant value exhibited by states of all the nodes in the system at a given time. If the maximally abundant value was not unique, one of the possibilities was chosen at random with equal probability. The autonomous field was spatially uniform, but its value may change as the system evolves. In the context of opinion or cultural models, this field may represent an endogenous global mass media influence that transmits the predominant opinion, cultural trend, or fashionable behavior present in a society.

We characterized the intensity of a global field by a parameter B∈[0,1] that expresses the probability of interaction of any agent with the field. Then, the probability of interaction between two agents is proportional to (1−B). On the other hand, the rewiring process in the network took place with a probability $P_{r}$, and we assumed that the node dynamics occurred with probability $1-P_{r}$. Thus, the node dynamics were coupled to the rewiring dynamics giving rise to a coevolutionary system. For the node state dynamics, we implemented a voter-like model that has been employed in coevolution of opinions and networks [43] and in various situations [44,45,46,47,48,49].

We built the initial random network with parameter values N=1000 and 〈k〉=4. Then, the states $g_{i}$ were assigned to the nodes at random with a uniform distribution. Therefore there were, on average, N/G agents in each state in the initial network. Here, we fixed the number of options at the value G=100.

Then, the coevolution dynamics of the system subject to a global field Φ, either external or autonomous, were defined by the following iterative algorithm:

1. Choose at random an agent *i* such that $k_{i}>0$.
2. With probability $P_{r}$, select at random an agent $j\in \nu_{i}$ and another agent $l\notin \nu_{i}$ such that $g_{i}=g_{l}$; remove the edge (i,j) and set the edge (i,l).
3. With probability $(1-P_{r})B$, set $g_{i}=g_{\Phi }$.
4. With probability $\left(1-P_{r}\right)\left(1-B\right)$, select at random an agent $m\in \nu_{i}$ and set $g_{i}=g_{m}$.
5. If Φ is autonomous, update the value $g_{\Phi }=\mathrm{mode}\left\{g_{i};\;i=1,2,\ldots ,N\right\}$

Step 2 specifies the rewiring process that modifies the network connectivity; new connections occur between agents with similar states. This rewiring decreased the number of links connecting nodes in different states, called active links. Links were rewired until a statistically stationary state, where the number of active links in the network dropped to zero, was reached. Steps 3 and 4 comprise the node imitation dynamics of the voter model: step 3 expresses the agent–field interaction, while step 4 describes the agent–agent interaction. In the case of an autonomous global field Φ, step 5 characterizes the time scale for the updating of state of the field $g_{\Phi }$. We verified that the collective behavior of this system was statistically similar if the steps of the node dynamics were performed before the rewiring process.

## 3. Results

In the absence of a global field (B=0), the imitation dynamics of the nodes increases the number of connected agents with equal states, while the rewiring process favors the segregation and fragmentation of the network [43]. Therefore, the evolution of the system eventually leads to the formation of a set of separate components, or subgraphs, disconnected from each other, with all members of a subgraph sharing the same state. Such subgraphs are called *domains*.

**Figure 1:**
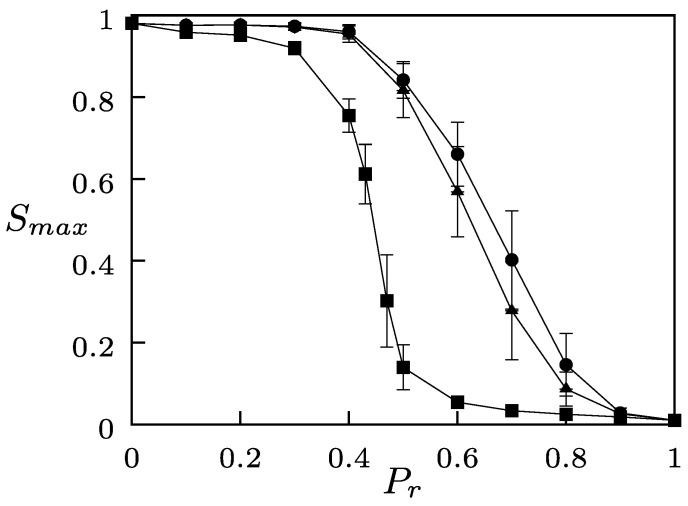
$S_{max}$ as a function of $P_{r}$ for the coevolutionary system subject to a global interaction field, for different values of the intensity *B*. The curves correspond to B=0 (squares); autonomous field with B=0.003 (triangles); external field with B=0.003 (circles). The parameters are G=100, N=1000, and 〈k〉=4. The error bars indicate standard error obtained over 100 realizations of random initial conditions for each value of $P_{r}$.

To characterize the collective behavior of the coevolutionary system subject to a global field, we used, as an order parameter, the normalized size of the largest domain in the system, averaged over several realizations of random initial conditions, denoted by $S_{max}$. Figure 1 shows the quantity $S_{max}$ as a function of the rewiring probability $P_{r}$ for different values of the intensity of the global field *B*. When no global fields were present (B=0, squares), our model reduced to the coevolution model of Holme and Newman [43] where, as $P_{r}$ increased, $S_{max}$ exhibited a transition at the critical value $P_{r}^{*}=0.458$, from a regime having a large domain whose size was comparable to the system size, characterized by values $S_{max}\to 1$, to a fragmented state consisting of small domains, for which $S_{max}\to 0$ [43].

Figure 1 indicates that, when a global field either external or autonomous was applied to the system, the fragmentation transition persisted, but the critical value of $P_{r}$ for which the transition took place increased as *B* incremented. The one-large domain phase consisted of agents sharing the state $g_{F}$ of the global field. For B>0, the critical value of $P_{r}$ in the presence of the external field was greater than the corresponding value for the autonomous field. As B→1, the fragmentation transition occurred at the value $P_{r}=1$ for either field. Thus, the presence of an external or an autonomous global field contributed to inhibit the fragmentation of the network.

**Figure 2:**
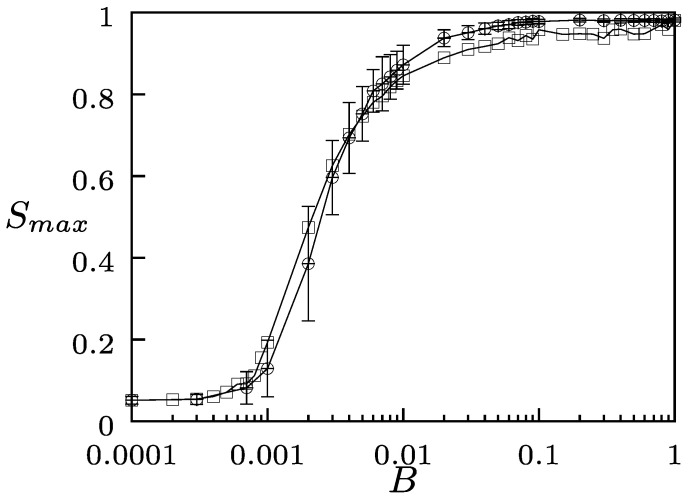
$S_{max}$ as a function of *B* (log scale) with fixed $P_{r}=0.6>P_{r}^{*}$. The curves correspond to an autonomous field (open circles) and to an external field (open squares). The parameters are G=100, N=1000, and 〈k〉=4. The error bars indicate standard errors obtained over 100 realizations of the initial conditions for each value of *B*.

In Figure 2, we show $S_{max}$ as a function of the intensity *B*, for both types of fields, with a fixed value of the rewiring probability $P_{r}=0.6>P_{r}^{*}$. For this value of $P_{r}$, the system reached a fragmented state when B=0. In Figure 2, we started from a fragmented state as the initial condition for each value of *B*. For small values of *B*, the system was fragmented in small domains, characterized by $S_{max}\to 0$. As the intensity *B* increased above some critical value, both the external and the autonomous fields produced a recombination of the network: the small domains possessing multiple states became united into one large domain whose elements shared the state $g_{\Phi }$ of the field. Thus, global interaction fields can have cohesive and homogenizing effects on a coevolutionary network.

**Figure 3:**
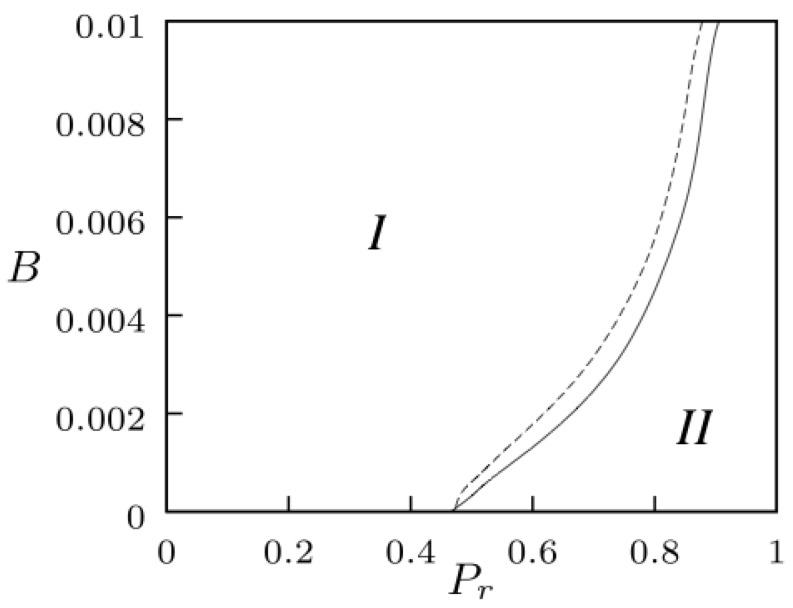
Critical boundaries for the fragmentation transition on the space of parameters $\left(B,P_{r}\right)$ for the adaptive system subject to an external global field (continuous line), or to an autonomous global field (dashed line). We calculate $S_{max}$ on the $\left(P_{r},B\right)$ plane with resolutions of $10^{-2}$ for $P_{r}$ and $10^{-3}$ for *B* and determine the critical values of $P_{r}$ and *B* by using finite size scaling analysis [43]. In each case, the boundary separates two phases: (*I*) a phase with one-large domain sharing the state of the global field; and (*II*) a fragmented phase with many small domains.

The critical values of *B* and $P_{r}$ for the fragmentation transitions in Figure 1 and Figure 2 were determined by using finite size scaling analysis, following the same approach proposed by Holme and Newman in [43]. Figure 3 displays the collective behavior on the space of parameters $\left(B,P_{r}\right)$ for the coevolutionary system subject to an external global field and to an autonomous global field. In each case, a critical boundary separated two phases: (*I*) an ordered phase characterized by $S_{max}\to 1$, where the network evolved to one large connected subgraph with all agents sharing the opinion of the field (above the curve); and (*II*) a fragmented phase for which $S_{max}\to 0$, where the system consisted of many small subgraphs with different opinions. An external global field appears a little more efficient than an autonomous field in preventing fragmentation in the coevolutionary system.

## 4. Discussion

We investigated the effects of the global interaction fields, external or autonomous, on an adaptive network of social agents with an opinion formation dynamics. The phase diagram in Figure 3 shows that the effects of both global fields on the collective behavior of the coevolutionary system were similar. The two phases arose from the competition between the homogenizing effect of the global field and the fragmentation of the network favored by the rewiring process. The similarity in the collective behaviors emerging in coevolutionary systems with external or autonomous global interaction fields signalled that the nature of the field, either external or endogenous, was qualitatively irrelevant. At the local level, the field acted effectively as an additional influential neighbor for every agent with the same node dynamics in each case.

In the context of dynamical systems, it has been shown that an analogy between an autonomous globally coupled system and a system subject to a global external drive can be established, because all the elements in each of these systems are affected by the corresponding global field in the same way at a given time [2]. Then, at the local level in either system, each element can be described as a drive–response dynamical system that can synchronize, and which eventually manifests as a collective state of synchronization. In social dynamics, a state of consensus can be interpreted as synchronization. In particular, for the coevolutionary opinion formation model considered, either external mass media or endogenous mass media trends induce consensus about their respective state. Our results suggest that the analogy between dynamical systems possessing external or autonomous global interaction fields can be extended to coevolutionary systems subject to global fields.

The opinion dynamics based on the simple imitation employed in this model led to the imposition of the states of the global mass media fields on the system. The imitation rule of voter dynamics has been applied to model elections, language competition, and clustering processes. We have found that a global field, either external or autonomous, can induce the recombination of a network broken in small domains into one large domain. In a social context, external mass media as well as the feedback of endogenous cultural trends can play a major role in preventing fragmentation and favoring cohesion in a society that possesses coevolution dynamics. Global mass media may contribute to control voters polarization and segregation in adaptive social networks. On the other hand, even under the influence of mass media, the existence of adaptive rewiring may counter the expected consensus and cohesion, as phase II on the phase diagram in Figure 3 shows.

Global mass media acting on systems possessing non-interacting states in their dynamics, such as in Axelrod’s model for cultural dissemination [52] or Deffuant’s bounded confidence model [53], can produce nontrivial effects other than imposing consensus, such as inducing disorder [12], alternative ordering [13], the emergence of chimera states [14], and promoting minority growth and polarization [15]. Future interesting extensions to be investigated include the influence of different node dynamics, such as those with non-interacting states, on the collective behavior of coevolutionary systems subject to global fields, general coevolution models [49], and the characterization of the topological properties of adaptive networks driven by global fields.

## Data Availability

Not applicable.
